# High prevalence and incidence of HSV-2 among people who inject drugs in Hai Phong, Vietnam, and risk factors associated with seroconversion

**DOI:** 10.1007/s10096-025-05079-8

**Published:** 2025-02-22

**Authors:** Morgana D’Ottavi, Ilenia Scialabba, Duong Thi Huong, Hoang Thi Giang, Pham Minh Khue, Vu Hai Vinh, Roselyne Vallo, Laurent Michel, Delphine Rapoud, Catherine Quillet, Nham Thi Tuyet Thanh, Juliette Bouniol, Khuat Thi Hai Oanh, Jonathan Feelmyer, Philippe Vande Perre, Didier Laureillard, Don Des Jarlais, Nicolas Nagot, Jean-Pierre Molès

**Affiliations:** 1 Pathogenesis and Control of Chronic and Emerging Infections, University of Montpellier, INSERM, EFS, Montpellier, 34394 France; 2 Faculty of Public Health, Hai Phong University of Medicine and Pharmacy, Hai Phong, Viet Nam, 04212 USA; 3 Infectious & Tropical Diseases Department, Viet Tiep Hospital, Viet Nam, Hai Phong, 04708 USA; 4 CESP UMR1018, Paris Saclay University, French Red Cross, Pierre Nicole center, Paris, 75005 France; 5 Supporting Community Development Initiatives, Viet Nam, Hanoi, 11513 Vietnam; 6 School of Global Public Health, New York University, New York, NY 10003 USA; 7 Pathogenesis and Control of Chronic and Emerging Infections, University of Montpellier, INSERM, EFS, CHU Montpellier, Montpellier, 34394 France; 8 Pathogenesis and Control of Chronic and Emerging Infections, University of Montpellier, INSERM, EFS, CHU Nîmes, Montpellier, 34394 France

**Keywords:** HSV-2, Prevalence, Incidence, Injecting drug users, Vulnerable population

## Abstract

**Purpose:**

Genital Herpes Simplex Virus-2 (HSV-2) epidemic is highly active worldwide and can be associated with severe morbidity and mortality. This study aimed to estimate the prevalence and incidence of HSV-2 infection among a vulnerable population of active heroin injectors in Hai Phong, Vietnam, and identify associated risk factors.

**Method:**

Associations between HSV-2 infection and socio-demographic characteristics and sexual behaviors were explored in a univariable analysis of seroprevalence. Risk factors were defined using a multivariable Poisson regression accounting for time of follow-up.

**Results:**

HSV-2 seroprevalence at baseline was 20.8% [95%CI: 17.8–22.2] for the 1281 men people who inject drugs (PWID), and 67.4% [95%CI: 60.1–74.1] for the 184 women PWID. For HSV-2 incidence, we accumulated a follow-up time of 1156.0 and 85.9 years for men and women, respectively. Standardised incidence rate was 4 [95%CI: 2.2–7.5] and 17.5 [95%CI: 5.7–53.8] infections per 100 person-years for men and women, respectively. Factors independently associated with HSV-2 seroconversion were HIV and injecting drug use for 5–10 years for men, and declared an uncontrolled HIV viral load and the use of street methadone.

**Conclusion:**

High HSV-2 prevalence and incidence among PWID in Hai Phong point out the burden of sexually transmissible infections in this population. Together these results advocate for a reinforcement of HSV-2 care and prevention in this population and identify PWID as future beneficiaries of upcoming therapeutic/prophylactic vaccines.

**Supplementary Information:**

The online version contains supplementary material available at 10.1007/s10096-025-05079-8.

## Introduction

Herpes simplex virus 2 (HSV-2) infection carries a large burden of morbidity worldwide, with variation according to population groups and their sexual behaviours. According to the WHO, an estimated 491.5 million people, or 13.2% of the world’s population aged 15–49 were HSV-2 seropositive in 2016 [1]. HSV-2 is the known cause of genital herpes, but a wide range of complications have also been associated with and attributed to HSV-2 infection, ranging from genital ulcer disease [2], meningitis [3], neurological morbidities [4], cervical cancer [5], and other sexually transmitted infections (STIs), most notably HIV [2, 6, 7, 8, 9, 10, 11].

In 2016 the WHO guidelines for the treatment of Genital Herpes Simplex Virus highlighted 3 key populations for HSV-2 treatment and prevention: female sex workers (FSW), men who have sex with men (MSM), and transgender persons [12]. In 2022, the WHO expanded their guidelines and included people who inject drugs (PWID) and people in prisons and other closed settings to their key populations [13].

PWID are part of key populations for STIs because injecting drug use is an established risk factor leading to riskier sexual behaviours [10]. The epidemiology of HSV-2 has been studied among PWID of high-income countries such as in New York [14], but unlike other key populations, there is little to no published literature regarding the prevalence or incidence of HSV-2 transmission among PWID in Southeast Asia, nor any characterization of the risk within this population. This gap needs to be filled as the Southeast Asian region has the largest concentration of drug users in the world [15]. Moreover, the efforts to prevent infectious diseases targeting PWID are currently focused quasi exclusively on harm reduction for injection-related risks [10], with the exception of self-declared FSW. It is important to describe the epidemiology of HSV-2 within the PWID population, especially considering the potential contribution of HSV-2 infection to HIV acquisition in this population, in which HIV infection is highly concentrated and implicates consequential financial and health burdens at the global scale [16, 17]. In addition, therapeutic and/or prophylactic vaccines are in the strategic plans of several institutions including NIH or WHO [18, 19], it is thus important to determine the extent to which this population should benefit from / be prioritised for these preventive interventions.

The DRug use and Infections in ViEtnam (DRIVE) project showed that a mass screening by peers from community based organizations (CBOs) combined with prevention and care for HIV could contribute to ending the HIV epidemic among PWID in Hai Phong, the third largest city in Vietnam [20, 21]. The study recruited participants by 4 consecutive respondent driven sampling surveys (RDSS) which allowed to enrol 70% of the estimated currently active PWID population, approximately 5000 persons. We took advantage of this study to estimate the prevalence and incidence of HSV-2 and to identify and describe PWID at higher risk for HSV-2 seroconversion, such that they might receive extra prevention activities in future screening programs in Hai Phong, Vietnam.

## Materials and methods

### Study population and design

The DRIVE project consisted in 5 RDSS: the first in 2014 (DRIVE-IN) which enrolled 603 PWID [22, 23], 2016 (RDSS1, *n* = 1383), 2017 (RDSS2, *n* = 1451), 2018 (RDSS3, *n* = 1445) and 2019 (RDSS4, *n* = 1268). The DRIVE study recruitment methods have previously been described in detail [24, 25]. Briefly, each RDSS started with 20 seeds previously selected by community workers. The seeds characteristics encompassed diversity regarding age, gender and HIV status. Equilibria were reached after 2 to 3 waves (median number of wave = 9) and homophilies were low for all major variables [20, 25]. Eligibility criteria for participation in any RDSS were being aged 18 and over, recent injection drug use (heroin or methamphetamine positive urinalysis and inspection for recent injection marks), living in Hai Phong, and ability to provide informed consent. In this observational sub-study, we included all DRIVE RDSS1 participants, for whom blood samples were stored at -80 °C. To account for the small number of women PWID, additional women who were enrolled in the DRIVE-IN RDSS, RDSS2, or RDSS3 were added and their baseline was the RDSS of the first HSV-2 serology. Transgender persons (*n* = 3) were classified as men for the purpose of the analyses. PWID could be recaptured as multiple participations across surveys and could be ascertained using fingerprinting. Additionally, all HIV positive participants from the first 3 RDSS (*n* = 740), and a sample of HIV negative participants (enrolled consecutively until completion of the sample size, *n* = 890) were invited to participate in a cohort study with follow-up every 6 months, up to M36.

### Data collection

At each visit (either RDSS or cohort follow-up), a structured interview was administered by a trained interviewer to collect declarative information related to socio-demographic characteristics, drug injection practices, mental health, drug and sexual behaviours.

### Blood collection and HSV-2 serology

At RDSS and follow-up visits, blood samples were collected in EDTA tubes, and plasma aliquots analysed for HIV/HCV serology, HIV viral load in PWID living with HIV, or stored for future analyses. Blood aliquots from baseline or the second visit were thawed only once for HSV-2 serological analysis. In case of multiple recaptures, the blood sample from the latest visit was tested. HSV-2 serology was determined using the HSV type 2 IgG ELISA kit (Kalon biological Ltd, Guildford, UK) following the manufacturers’ recommendations. The kit classified the serology into negative, undetermined, or positive. We used a conservative criterion for positivity i.e. all samples identified as positive and those identified as undetermined at inclusion but which turn positive at a following visit. As per manufacturer’s recommendation, a second positive test identifies a primary infection in progress at the time of the first test.

### Statistical analysis

Baseline characteristics were presented as frequencies and percentages for categorical variables, and as medians and interquartile ranges (IQR) for continuous variables. Characteristics were stratified by gender, as well as by baseline HSV-2 serology. Differences in baseline characteristics according to HSV-2 serology were tested using Pearson’s Chi-squared test, or Fischer’s Exact test if conditions were not met, and Wilcoxon’s rank sum test for continuous variables. For ordinal variables, a chi-square statistic for linear trend was also calculated.

Global HSV-2 seroprevalence was calculated using only participants that were tested for HSV-2 at the time of RDSS1, and adjusted for the sampling design [26]. All subsequent analyses were stratified by gender. Seroprevalence measures were presented with their 95% Pearson exact confidence intervals (CI).

Incidence data was calculated among HSV-2 seronegative PWID at baseline who had at least one other visit, either during cohort follow up or at a subsequent RDSS. Time to infection was defined as mid-time between baseline and an incident HSV-2 positive serological test. A direct standardization by age group was applied to incidence rate calculations, using the initial RDSS1 age structure as the reference population, and to account for differences in participants that returned versus those that were HSV-2 seronegative at baseline but were not tested again (compared characteristics available in Supplementary Table S2). Standardised incidence rates were presented per 100 person-years (PY), with their 95% Poisson CI.

We identified factors associated with HSV-2 seroconversion, according to the baseline characteristics of participants, using multivariable Poisson regression accounting for time of follow-up. Parameter selection was based on results obtained in a first univariable analysis step (Supplementary Table S3) and a second backwards stepwise selection, both using *p* < 0.20 as a threshold. Final models were created based on the parameter selection and optimization of the Akaike information criterion (AIC). All multivariable models were age-adjusted. This was done as an exploratory analysis and models are highly fitted to the specific data herein.

The threshold for statistical significance was set at *p* < 0.05. All statistical analyses were carried out using Stata 16.1 (Stata Corp, College Station, Texas).

## Results

**Fig. 1:**
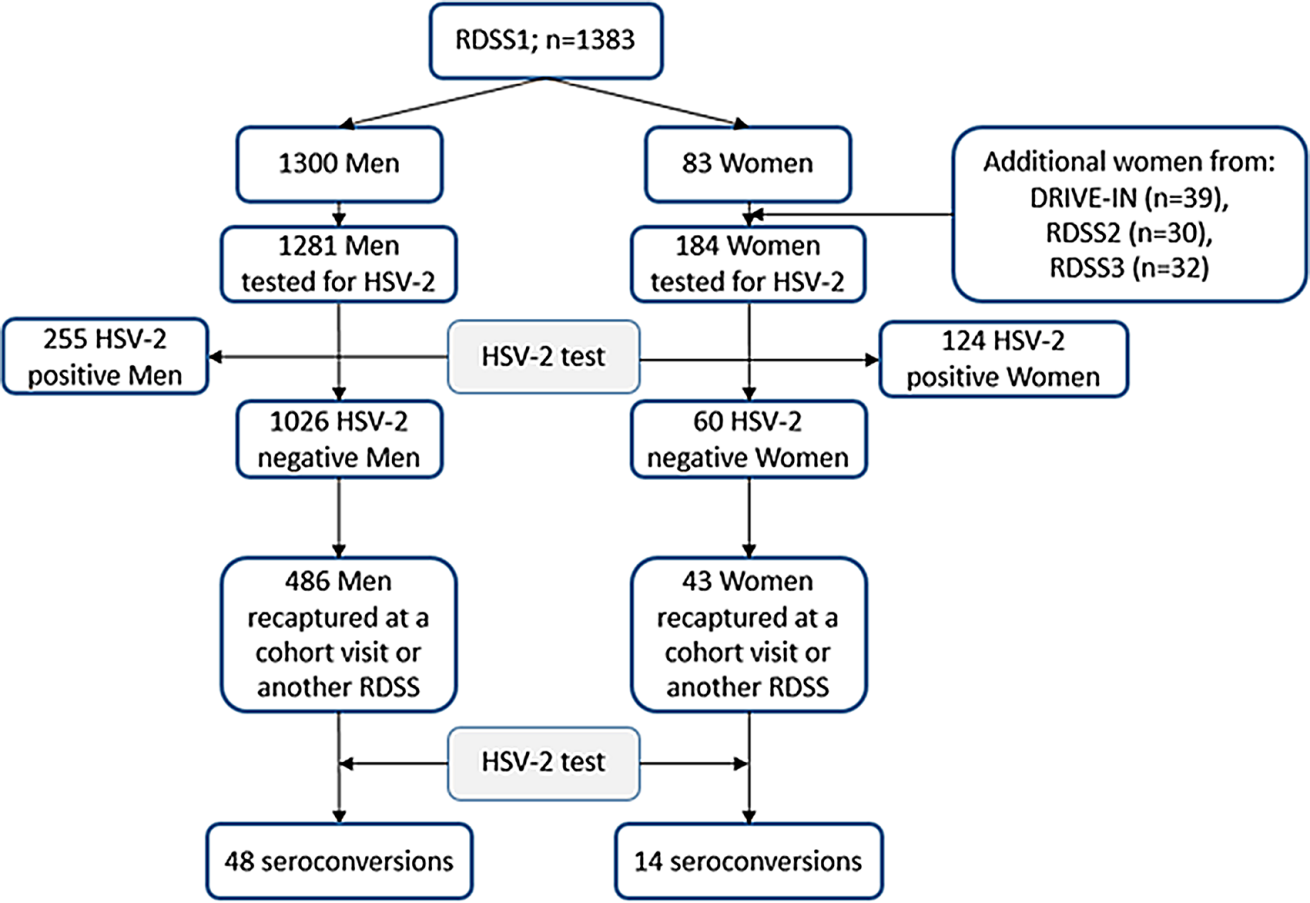
Participant flowchart. Abbreviations: RDSS, Respondent driven sample survey; HSV-2, Herpes simplex virus 2; DRIVE, DRug use and Infections in ViEtnam

Of the 1383 PWID included in the first RDSS, we obtained blood samples for 1364 participants, 83 of whom were women. An additional 101 women were included from a previous RDSS done as formative research, the second and third RDSS (Fig. 1).

The median age of participants was 39 [33–45] years for men and 35 [30–40] years and women (Supplementary Table S1). Among men, all had been injecting heroin for at least 1 year, and a quarter (24.6%) declared over 15 years of injection. During the last month, the median frequency of injections was 2 [IQR: 2–3] per day. Among women, 40% had been injecting heroin for less than 5 years while 12.8% had been injecting for over 15 years. The median frequency of injection for women was 3 times per day [IQR: 2–3] over the last month. Furthermore, 11.9% and 14.3% of men and women, respectively, reported being treated with methadone provided by the national methadone maintenance treatment program (Supplementary Table S1). Roughly one-third of included men (29.8%) and women (31.0%) were HIV seropositive.

### HSV-2 Seroprevalence at baseline

**Fig. 2:**
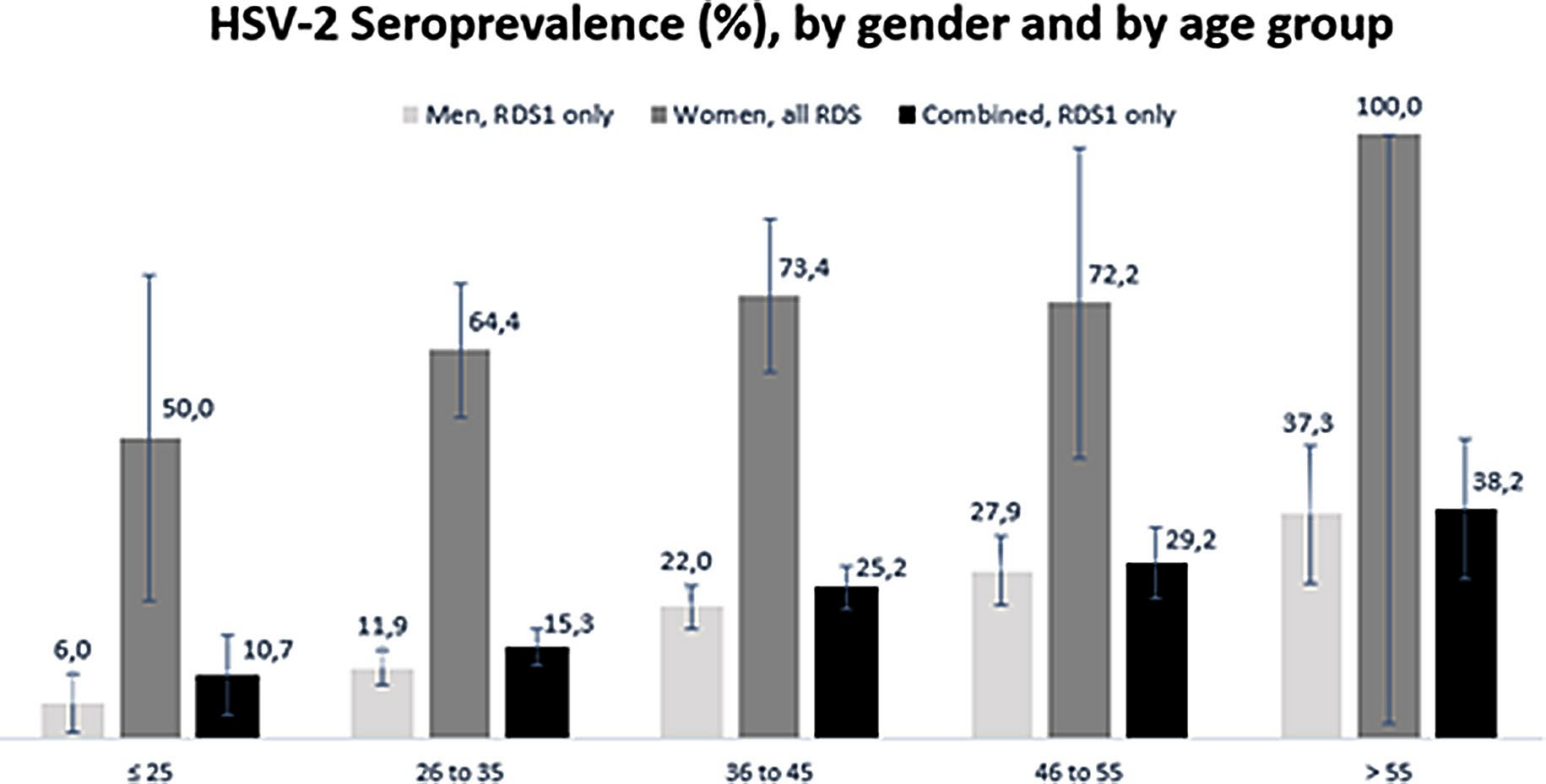
HSV-2 seroprevalence at baseline, by gender and by age group. Abbreviations: RDSS, Respondent driven sample survey; HSV-2, Herpes simplex virus 2. Men: *n* = 3 missing values; p-value for linear trend < 0.0001. Women: p-value for linear trend = 0.0739 (*p* = 0.1497 with 46–55 & 55 + category combined). Combined: *n* = 3 missing values; p-value for linear trend < 0.0001

Among the 1364 PWID, the sampling design adjusted HSV-2 seroprevalence was 23.6% [95%CI: 20.5–26.4]. Global HSV-2 seroprevalence increased significantly with age (*p* < 0.001) (Fig. 2). When stratified by gender, adjusted HSV-2 seroprevalence was 20.8% [*n* = 1281; 95%CI: 17.8–23.8] for men and 69.4% [*n* = 83; 95%CI: 37.5–91.0] for women. Among all women enrolled in this work (*N* = 184), the HSV-2 seroprevalence was 67.4% [95%CI: 60.1–74.1]. Of note, equilibrium for HSV-2 seropositivity was reached at the third wave.

### Baseline characteristics by HSV-2 serology and gender

HSV-2 seropositive men tended to be less educated than seronegative men (Table 1). HIV prevalence, HCV co-infection, and drug use patterns were similar among HSV-2 seronegative and seropositive men (data not shown).

Seropositive women had higher income than seronegative women with 31.5% versus 15.0%, respectively (p-value for linear trend = 0.033). HSV-2 seropositive women were equally HIV positive (27.4%) compared to HSV-2 seronegative women (38.3%; *p* = 0.133) (Table 1).

Seropositive and seronegative PWID declared similar number of partners, use of condoms, and rates of sex in exchange for money, as a client or as a sex worker but also the same proportion of seropositive and seronegative PWID declared never having sexual intercourse in their lifetime (Table 1).

**Table 1:** Adjusted socio-demographic characteristics and declared sexual behaviours at baseline, by gender and by HSV-2 serology (in % or med [IQR])

Characteristics	Men (RDS1; *n* = 1281)			Women (all; *n* = 184)		
	HSV-2 neg. (*n* = 1026)	HSV-2 pos. (*n* = 255)	*p*-value	HSV-2 neg. (*n* = 60)	HSV-2 pos. (*n* = 124)	*p*-value
Age	37 [32–44]	43 [37–48]	< 0.001^µ^ °	32 [28-38.5]	35 [31–41]	0.035°
Education level			0.079^µ^†			0.240†
*None*	2.5	4.7		8.3	14.5	
*Primary or middle*	67.1	69.3		78.3	75.0	
*High school or +*	30.4	26.0		13.3	10.5	
Marital Status			0.410^µ^			0.115
*Single*	36.7	32.7		25.0	16.9	
*Married/cohabitating*	36.3	37.0		41.7	33.9	
*Divorced*,* or widowed*	27.0	30.3		33.3	49.2	
Is a registered resident of the city	89.8	91.7	0.364^µ^	76.7	62.9	0.062
Has health insurance	25.0	27.6	0.402^µ^	23.3	21.8	0.812
Monthly income			0.285^µ^†			0.033°†
*0 to < 3 M VND*	20.2	26.4		18.3	12.9	
*3 to < 6 M VND*	45.8	39.8		41.7	33.9	
*6 to < 9 M VND*	21.2	20.9		25.0	21.8	
*≥ 9 M VND*	12.8	13.0		15.0	31.5	
Sex for money as a source of income	0.9	1.2	0.715^µ^*	31.7	37.9	0.408
Having sexual intercourse ever	55.2	51.8	0.330^λ^	83.3	79.0	0.491
Number of partners ††			0.216^φ^*			0.116
*None*	52.8	54.0		40.7	44.4	
*1 to 10*	47.3	45.6		44.1	29.9	
*More than 10*	0.0	0.4		15.3	25.6	
Uses condoms during last sexual encounter^α^	40.9	33.1	0.099^µ^	30.6	31.9	0.879 ^τ^
Engaged in at-risk sex behaviour ^αΎ††^	66.7	72.3	0.220^µ^	89.8	95.6	0.277^θ^*
Has paid for sex ††^α^	25.7	21.4	0.301	2.0	0.0	0.338*
and has engaged in at-risk behaviour with sex worker(s)^β^	24.1	10.7	0.140*	0.0	N.A.	N.A.
Has received money in exchange for sex ^†† α^	3.2	2.3	0.780*	44.0	54.1	0.246
and has engaged in at-risk behaviour with client(s)^β^	38.9	0.0	0.521*	22.7	18.9	0.755*
HIV seropositive	29.2 ^**θ**^	32.4 ^**θ**^	0.317	38.3	27.4	0.133
And has viral load *> 1000 cp/mL*	23.7 ^**µ**^	22.2 ^**ρ**^	0.788	47.6 ^**θ**^	31.0 ^**π**^	0.233
HCV seropositive	70.0 ^**θ**^	73.1 ^**θ**^	0.332	65.0	65.3	0.966

Adjustment accounted for RDSS weights. Abbreviations: RDSS, Respondent Driven Sampling Survey; HSV-2, Herpes simplex virus 2; VND, Viet Nam Dong; HIV, Human immunodeficiency virus; cp/mL, copies per millilitres; HCV, Hepatitis C virus. †p-value for linear trend, * using Fischer’s Exact test, †† in the past 3 months; ‡ composite variable if answered “yes” to has ever smoked meth or has ever injected meth, and declares smoking or injecting at least once in past month or MET was present in urine. ^α^ Frequencies based on those who responded “yes” to having had sexual intercourse at least once in their lifetime. ^Ύ^At-risk behaviour defined as either not having used condoms during last sexual encounter, reporting never or occasionally using condoms; ^β^At-risk behaviour defined as either not having used condoms during last sexual encounter, reporting never, occasionally, or almost always using condoms. ^µ^ 3 missing values, ^λ^ 6 missing values, ^ε^ 4 missing values, ^θ^ 2 missing values, ^δ^ 41 missing values, ^ρ^ 1 missing value, ^Δ^ 7 missing values, ^φ^ 9 missing values, ^τ^ 8 missing values, ^π^ 5 missing values

### Standardised incidence rate for HSV-2 seroconversion

Overall, of the 1086 HSV-2 seronegative PWID at baseline, about half of the men were recaptured at a cohort follow-up visit or another RDSS (486/1026), and more than two thirds of the women (43/60). Recaptured men and women differed from those that did not return and did not have a second serologic sample taken (Supplementary Table S2). The recaptured men were older, had been injecting heroin for more years and had more health insurance coverage. Fewer were HIV positive, but among those that were HIV positive, those that were recaptured were more likely to have a detectable HIV viral load. Recaptured women were also older and had higher health insurance coverage. Overall, we accumulated a follow-up time of 1156.0 and 85.9 years, and 48 and 14 seroconversions for men and women, respectively. The raw incidence rate was of 5 new cases per 100PY [95%CI: 3.8–6.4] for both men and women combined, of 4.2/100PY [95%CI: 3.1–5.5] among men and of 16.3/100PY [95%CI: 8.9–27.3] among women. To account for the younger age of loss-to-follow up and highly heterogeneous rate of acquisition across age groups, standardised incidence rates were calculated; the overall incidence rate was of 5/100PY [95%CI: 1.4–8.7], of 4/100PY [95%CI: 2.3–7.5] among men and of 17.5/100PY [95%CI: 5.7–53.8] among women.

There was no newly acquired HIV-infection during follow-up.

### PWID at high risk of HSV-2 seroconversion

Multivariable analysis of HSV-2 seroconversion showed that PWID men who were HIV positive at baseline had a 2.2 higher incidence rate [95%CI: 1.2-4.0] than those who were HIV negative, independently of age, income, and injection duration (Table 2). PWID men that had been injecting for 5–10 years were also 2.4 times more at risk of seroconversion (Table 2).

**Table 2:** Risk factors for HSV-2 seroconversion for men PWID for whom a second HSV-2 test was performed (*N* = 485)

Characteristic at baseline	Crude IRR [95%CI]	aIRR [95%CI]	*p*-value
HIV status			
*Positive*	1.9 [1.1–3.4]	2.2 [1.2-4.0]	0.010
Having been injecting heroine			
*< 5 years*	Ref.	Ref.	
*5 to < 10 years*	1.7 [0.9–3.4]	2.4 [1.2–4.8]	0.014

Multivariable model is also adjusted for age and income. Abbreviations: HSV-2, Herpes simplex virus 2; PWID, People who inject drugs; HIV, Human immunodeficiency virus

For women, multivariable analysis put forth reporting sex for money at baseline as a risk factor for of HSV-2 seroconversion, increasing incident HSV-2 risk almost 4-fold, independently of age and income (Table 3). Furthermore, a positive HIV status with an uncontrolled viral load (exceeding 1000 cp/mL) was also found to be a major risk factor of HSV-2 seroconversion (IRR 9.2, [95%CI: 2.0–43.0], *p* = 0.005), independently of age, income, and reported sex for money (Table 3). Meanwhile the use of street methadone, reduced the risk of incident HSV-2 (IRR 0.14, [95%CI: 0.02–0.92], *p* = 0.041) (Table 3). In a sensitivity analysis, we added the variable “no sexual activity ever” in the multivariable models for both men and women (Supplementary Table S4). This change did not alter the outputs of the final models, neither in terms of parameter selection nor in the direction or effect size of observed associations, except for HIV viral load in the model for women, which is no longer significantly associated with seroconversion (Supplementary Table S4).

**Table 3:** Risk factors for HSV-2 seroconversion for women PWID for whom a second HSV-2 test was performed (*n* = 37 in the final model, 6 missing values for street methadone, of which 1 also has HIV VL missing)

Characteristic at baseline	Crude IRR [95%CI]	IRR [95%CI]	*p*-value
Reported sex for money	2.4 [0.8-7.0]	3.8 [1.0-14.9]	0.057
HIV status			
*HIV negative*,* or HIV positive with viral load < 1000 cp/mL*	Ref.	Ref.	
*HIV positive with viral load > 1000 cp/mL*	3.9 [1.3–11.6]	9.2 [2.0–43.0]	0.005
Street methadone		0.14 [0.02–0.92]	0.041

Multivariable model is adjusted for age and income. Abbreviations: HSV-2, Herpes simplex virus 2; PWID, People who inject drugs; HIV, Human immunodeficiency virus; cp/mL, copies per millilitres

## Discussion

This study proposed a comprehensive epidemiological picture of HSV-2 among a representative population of PWID from Hai Phong, Vietnam. Our findings showed high HSV-2 seroprevalence (67.4%; 95%CI: 60.1–74.1) among women, over 3-fold that observed among men (20.8%; 95%CI: 17.8–23.8). The standardised incidence rate was more than 4-fold higher among women than among men which peaked at 17.5 per 100PY [95%CI: 5.7–53.8]. These rather unique observations illustrated the burden of HSV-2 infection and reflected an active epidemic within this population.

Among men, the HSV-2 prevalence described herein was similar to the 19.4% [95%CI: 12.1–27.9] previously reported in the general population in Vietnam, and the 23.6% [95%CI: 20.9–26.3] group-specific seroprevalence for MSM, MSWs, or transgender persons of for all Asian countries [27]. The seroprevalence reported herein was also comparable to the 22% among PWID in Northern Vietnam reported by Go et al. [28]. The HSV-2 seroprevalence among women was comparable to the FSW group-specific seroprevalence of 62.2% [95%CI: 58.9–65.6] for all Asian countries [27], as well as the 58.3% and 79% from FSW studies from the Vietnam-China border and Singapore, respectively [29, 30]. It was, however, much higher than the estimated seroprevalence of 8.8% in Hanoi and 30.8% in Ho Chi Minh City (HCMC) among married women aged 15–69 years in the general population [31]. CBO members reported that a vast majority of women PWID in Hai Phong were involved in sex work (personal communication), which our results corroborate.

Furthermore, we reported an incidence rate in men PWID of 4 new infections per 100PY, largely above the 0.5% incident infections for men in a worldwide meta-analysis, and the 2.6 per 100PY in healthy adult men – the highest incidence rate reported so far in Asia, from an Indian cohort study [16, 27]. A quarter of the men PWID with an HSV-2 seronegative test declared to “have been engaged in at-risk behaviour with sex worker”. Given the HSV-2 prevalence among Vietnamese FSW and PWID FSW [32, 33], this high incidence likely stems from these exchanges. The 17.5 per 100PY incidence rate for women PWID (1 in 6) was consistent with the 21.9 reported for FSW in China [34]. Given that STI never come alone [35], the poor knowledge of these infections and their current underdiagnoses could also represent a risk factor explaining such high incidence. However, this aspect was not addressed in the present study. Improved epidemiological data and reinforced access to STIs screening and care among PWID may be a key factor in controlling incident HSV-2 in the general population in Vietnam.

Given the route of HSV-2 transmission, a declaration bias was clearly identified as evidenced by the baseline 48% and 21% of HSV-2 seropositive men and women, respectively, that declared never having had sexual intercourse in their lifetime. In addition, 15% of men and 21% of women who seroconverted also declared no sexual activity in their lifetime at the time of the HSV-2 positive test. Nonetheless, in a sensitivity analysis we used this misdeclaration as a potential identifier of persons at-risk of seroconversion, arguing that this type of disability reflects a more general embarrassment leading to risky sexual behaviour. In the multivariable regressions done in this sensitivity analysis, misdeclaration by men was not a potential identifier of seroconversion, whereas it was for women (IRR 16.4 [95%CI: 3.1–89.4], *p* = 0.001). To our knowledge, such observation has not been reported so far, and deserves furthered attention to confirm this link in other contexts.

FSW clients such as PWID involved in transactional and/or commercial sex, are likely the bridging population for STIs transmissions in Vietnam, as already reported in others countries [32]. In-depth interviews among young people in HCMC and a study on women in Northern Vietnam both found a general lack of knowledge regarding STIs symptoms, causes, and condom use[35]. Furthermore, women had a notable passiveness when it came to decision making for condom use[35]. Overall, most FSW sought treatment at pharmacies when they noticed symptoms of the genital tract, but care-seeking behaviours within health facilities and regularity of HSV-2 testing is hampered by elevated treatment costs, prejudiced and/or stigmatizing attitudes of service providers and a concern for confidentiality, and a lack of information concerning testing services [32, 33]. A strong correlation has also been observed between drug use and condom slippage/breakage, emphasizing the importance of proper sexual education among PWID [13]. PWID have demonstrated high-risk perceptions of HIV, but limited knowledge or concern for other STIs [10]. Although they have adopted injection practices that greatly reduce the risk of HIV transmission, such as HIV positive PWID injecting last during the back loading injection session (CBO communication), they are not aware of combined risks such as HSV-2 coinfection. Nevertheless, the recent high antiretroviral coverage among PWID contributes to an important reduction of this risk, and may explain why we did not observe any HIV seroconversions among initially HSV-2 seronegative PWID. Prevention measures should encompass risks regarding injection practices, as well as at-risk sexual behaviours.

Our study has a number of limitations. First, this analysis used data from RDSS. Though this unique type of sampling allows recruiting large numbers of persons in hidden key populations in a short period of time, the obtained sample may be subject to a selection bias. We believe this bias was limited in our analysis by correcting for the sampling design in prevalence calculations and by applying a direct standardization for age for incidence rate calculations. Secondly, our results reflect an important declarative bias with regards to the face-to-face questionnaire and sexual behaviours. Although precautions were taken to reduce stigma during the RDS surveys (implemented within CBO offices, presence of FSW CBO members), this is an indication that face-to-face questionnaires may not be the ideal method for accurately collecting this type of sensitive information, or may not be sufficient as a stand-alone tool for assessing at-risk behaviours. Thirdly, we analysed the result of the assay with a strict definition for HSV-2 seropositivity, as indeterminate results were considered negative. Our conclusions should be subsequently considered as conservative. Finally, our findings are specific to Vietnam and cannot be extrapolated to other settings as such.

## Conclusion

Our findings highlight high prevalence and incidence of HSV-2 infection among PWID in Hai Phong, especially among women. These results plead for sensitization on genital herpes among PWID, including the recognition of symptoms and the reinforcement of care seeking behaviours for recurrent herpes episodes, and raise concerns on the burden of other STIs. This population should not be neglected when therapeutic/prophylactic vaccines will become available.

## Electronic supplementary material

Below is the link to the electronic supplementary material.

Supplementary Material 1

Supplementary Material 2

Supplementary Material 3

## Data Availability

The data that support the findings of this study are available from the corresponding author upon reasonable request.
