# Supplementary material for: High prevalence and incidence of HSV-2 among people who inject drugs in Hai Phong, Vietnam, and risk factors associated with seroconversion
Source: Eur J Clin Microbiol Infect Dis. 2025 Feb 22;44(5):1119–27. doi: 10.1007/s10096-025-05079-8 (PMC12062056; doi:10.1007/s10096-025-05079-8)
Supplement: Supplementary file 3 — Supplementary Material 3 [file 10096_2025_5079_MOESM3_ESM.docx]

**Supplementary Materials**

High prevalence and incidence of HSV-2 among people who inject drugs in Hai Phong, Vietnam, and risk factors associated with seroconversion.

**European Journal of clinical microbiology & infectious diseases**

Morgana D’Ottavi^1§^, Ilenia Scialabba^1§^, Duong Thi Huong^2^, Hoang Thi Giang^2^, Pham Minh Khue^2^, Vu Hai Vinh^3^, Roselyne Vallo^1^, Laurent Michel^4^, Delphine Rapoud^1^, Catherine Quillet^1^, Nham Thi Tuyet Thanh^5^, Juliette Bouniol^1^, Khuat Thi Hai Oanh^5^, Jonathan Feelmyer^6^, Philippe Vande Perre^7^, Didier Laureillard^8^, Don Des Jarlais^6^, Nicolas Nagot^7^* & Jean-Pierre Molès^1^*

^1^Pathogenesis and control of chronic and emerging infections, University of Montpellier, INSERM, EFS, 34394 Montpellier, France.

^2^Faculty of Public Health, Hai Phong University of Medicine and Pharmacy, 04212 Hai Phong, Viet Nam.

^3^Infectious & Tropical Diseases Department, Viet Tiep Hospital, 04708 Hai Phong, Viet Nam.

^4^CESP UMR1018, Paris Saclay University, Pierre Nicole center, French Red Cross, 75005 Paris, France.

^5^Supporting Community Development Initiatives, 11513 Hanoi, Viet Nam.

^6^School of Global Public Health, New York University, New York, NY 10003, USA.

^7^Pathogenesis and control of chronic and emerging infections, University of Montpellier, INSERM, EFS; CHU Montpellier, 34394 Montpellier, France.

^8^Pathogenesis and control of chronic and emerging infections, University of Montpellier, INSERM, EFS; CHU Nîmes, 34394 Montpellier, France.

*** Correspondence:**Jean-Pierre Molès
[jean-pierre.moles@inserm.fr](mailto:jean-pierre.moles@inserm.fr)

**Supplementary Table S1. Adjusted socio-demographic characteristics at baseline, by gender**

| Characteristic | Men (RDS1; N=1281)  % or Med [IQR] | Women (all; N=184)  % or Med [IQR] |
| --- | --- | --- |
| Age | 39 [33-45]^α^ | 35 [30-40] |
| Age, in categories  *≤ 25*  *26 to 35*  *36 to 45*  *46 to 55*  *> 55* | 3.9^α^  33.0  40.1  17.7  5.2 | 7.6  47.3  34.8  9.8  0.5 |
| Education level  *None*  *Primary or middle*  *High school or higher* | 3.0^α^  67.5  29.5 | 12.5  76.1  11.4 |
| Marital Status  *Single*  *Married/cohabitating*  *Divorced, separated, or widowed* | 35.9^α^  36.5  27.6 | 19.6  36.4  44.0 |
| Having city registration | 90.2 | 67.4 |
| Having health insurance | 25.5^α^ | 22.3 |
| Monthly income  *0 to < 3M VND*  *3 to < 6M VND*  *6 to < 9M VND*  *≥ 9M VND* | 21.4^α^  44.6  21.1  12.8 | 14.7  36.4  22.8  26.1 |
| Having sex for money | 0.9^α^ | 35.9 |
| Number of years of heroin injection  *<5 years*  *5 to <10 years*  *10 to <15 years*  *≥15 years* | 28.2^β^  25.7  21.6  24.6 | 40.0^γ^  29.4  17.8  12.8 |
| Frequency of heroin injection on a typical day during the past month | 2 [2-3]^β^ | 3 [2-3]^δ^ |
| Methamphetamine user‡ | 48.8^β^ | 60.1^ε^ |
| Methadone in urine | 42.9^γ^ | 25.7^θ^ |
| Currently receiving methadone maintenance treatment | 11.9^β^ | 14.3^δ^ |
| Using street methadone | 37.3^λ^ | 30.8^ε^ |
| Positive HIV serology | 29.8^γ^ | 31.0 |
| Having Viral Load *>1000 cp/mL* | 23.3^α^ | 38.0^μ^ |
| Positive HCV serology | 70.6^γ^ | 65.2 |

Adjustment accounted for RDSS weights. Abbreviations: RDS, Respondent Driven Sampling; HSV-2, Herpes simplex virus 2; VND, Viet Nam Dong; HIV, Human immunodeficiency virus; cp/mL; copies per milliliters; HCV; Hepatitis C virus. °Statistically significant, †p-value for linear trend, * using Fischer’s Exact test, ‡ composite variable if answered “yes” to has ever smoked meth. or has ever injected meth., and declares smoking or injecting at least once in past month or meth. was present in urine. ^α^ 3 missing values, ^β^ 6 missing values, ^γ^4 missing values, ^δ^ 2 missing values, ^ε^ 41 missing values, ^θ^ 1 missing value, ^λ^ 8 missing values, ^μ^ 7 missing values.

**Supplementary Table S2. Comparative characteristics of participants that were recaptured for a second HSV-2 serological test versus those that were not, by gender**

| Characteristics | HSV-2 negative Men (N=1026)  % or Med [IQR] | | | HSV-2 negative Women (N=60)  % or Med [IQR] | | |
| --- | --- | --- | --- | --- | --- | --- |
|  | Recaptured  *N=486* | Lost to FU  *N=538^Ύ^* | p-value | Recaptured  *N=43* | Lost to FU  *N=17* | p-value |
| Age | 40 [35-45] | 35 [30-43] | <0.001° | 34 [29-40] | 27 [25-33] | 0.007° |
| Age, in categories  *≤ 25*  *26 to 35*  *36 to 45*  *46 to 55*  *> 55* | 1.7  28.4  48.4  17.1  4.5 | 7.3  43.5  30.7  14.9  3.7 | <0.001° | 4.7  48.8  37.2  9.3 | 29.4  58.8  5.9  5.9 | 0.010*° |
| Education level  *None*  *Primary or middle*  *High school or higher* | 2.1  66.9  31.1 | 3.0  67.3  29.7 | 0.607 | 9.3  79.1  11.6 | 5.9  76.5  17.7 | 0.876* |
| Marital Status  *Single*  *Married/cohabitating*  *Divorced, separated, or widowed* | 37.5  35.2  27.4 | 35.3  38.1  26.6 | 0.615 | 46.5  23.3  30.2 | 29.4  29.4  41.2 | 0.474* |
| Having a city registration | 93.8 | 86.3 | <0.001° | 76.7 | 76.5 | 1.000* |
| Having health insurance | 30.7 | 20.0 | <0.001° | 30.2 | 5.9 | 0.050*° |
| Monthly income  *0 to < 3M VND*  *3 to < 6M VND*  *6 to < 9M VND*  *≥ 9M VND* | 19.8  47.3  22.2  10.7 | 20.6  44.4  20.3  14.7 | 0.241 | 23.3  39.5  27.9  9.3 | 5.9  47.1  17.7  29.4 | 0.133* |
| Sex for money as a source of income | 0.8 | 0.9 | 1.000* | 30.2 | 35.3 | 0.763* |
| Number of years of heroin injection  *<5 years*  *5 to <10 years*  *10 to <15 years*  *≥15 years* | 20.2†  26.4  22.3  31.1 | 39.1†  26.8  19.6  14.5 | <0.001° | 44.2  27.9  20.9  7.0 | 47.1  23.5  11.8  17.7 | 0.596* |
| Frequency of heroine injection on a typical day during the past month | 2 [2-3]† | 2 [2-3]† | 0.468 | 2 [2-3] | 3 [2-3] | 0.469 |
| Methamphetamine user^1^ | 46.5 | 52.8 | 0.045° | 44.2 | 29.4 | 0.385* |
| Methadone in urine | 45.1 | 40.5 | 0.142 | 25.6 | 35.3 | 0.530* |
| Use street methadone | 36.4 | 37.3 | 0.771 | 18.9 | 42.9 | 0.322* |
| HIV seropositive | 43.8 | 16.0 | <0.001° | 39.5 | 35.3 | 1.000* |
| Viral Load >1000 cp/mL | 16.2‡ | 41.9 | <0.001° | 43.8† | 60.0† | 0.635* |
| HCV seropositive | 75.1 | 65.4 | 0.001° | 67.4 | 58.8 | 0.528 |

Abbreviations: RDSS, Respondent Driven Sampling Survey; HSV-2, Herpes simplex virus 2; VND, Viet Nam Dong; HIV, Human Immunodeficiency Virus; cp/mL; copies per milliliters; HCV; Hepatitis C Virus. *using Fischer’s Exact test, ^1^composite variable if answered “yes” to has ever smoked meth or has ever injected meth, and declares smoking or injecting at least once in past month or MET was present in urine. ^Ύ^2 missing values for the entire column. †1 missing value, ‡3 missing values.

**Supplementary Table S3. Comparison of baseline characteristics among those that have a 2^nd^ HSV-2 test, by gender and by HSV-2 seroconversion**

| Characteristics | Men (N=486) % or Med [IQR] | | | Women (N=43) % or Med [IQR] | | |
| --- | --- | --- | --- | --- | --- | --- |
|  | 2^nd^ HSV-2 test | |  | 2^nd^ HSV-2 test | |  |
|  | Neg.  N= 438 | Pos.  N= 48 | p-value | Neg.  N= 29 | Pos.  N= 14 | p-value |
| Age | 40 [34-45] | 40 [36-46] | 0.435 | 36 [31-40] | 33 [29-39] | 0.577 |
| Age, in categories  *≤ 25*  *26 to 35*  *36 to 45*  *46 to 55*  *> 55* | 1.8  28.8  48.2  16.2  5.0 | 0.0  25.0  50.0  25.0  0.0 | 0.303 | 3.5  44.8  37.9  13.8 | 7.1  57.1  35.7  0.0 | 0.472 |
| Education level  *None*  *Primary or middle*  *High school or higher* | 1.8  67.8  30.4 | 4.2  58.3  37.5 | 0.202 | 13.8  72.4  13.8 | 0.0  92.9  7.1 | 0.367 |
| Marital Status  *Single*  *Married/cohabitating*  *Divorced, separated, or widowed* | 35.2  36.5  28.3 | 35.4  45.8  18.8 | 0.294° | 24.1  48.3  27.6 | 21.4  42.9  35.7 | 0.915 |
| Having a city registration | 93.8 | 93.8 | 1.000 | 75.9 | 78.6 | 1.000 |
| Having health insurance | 29.7 | 39.6 | 0.187 | 31.0 | 28.6 | 1.000 |
| Monthly income  *0 to < 3M VND*  *3 to < 6M VND*  *6 to < 9M VND*  *≥ 9M VND* | 20.5  46.3  21.9  11.2 | 12.5  56.3  25.0  6.3 | 0.361 | 17.2  44.8  27.6  10.3 | 35.7  28.6  28.6  7.1 | 0.596 |
| Sex for money as a source of income | 0.9 | 0.0 | 1.000 | 17.2 | 57.1 | 0.013* |
| Number of years of heroin injection  *<5 years*  *5 to <10 years*  *10 to <15 years*  *≥15 years* | 20.1†  26.1  23.3  30.4 | 20.8  29.2  12.5  37.5 | 0.344 | 48.3  31.0  13.8  6.9 | 35.7  21.4  35.7  7.1 | 0.418 |
| Frequency of heroine injection on a typical day during the past month | 2 [2-3]† | 2 [1.5-3] | 0.593 | 2 [2-3] | 3 [2-3] | 0.969 |
| Methamphetamine user^1^ | 46.6 | 45.8 | 0.922° | 44.8 | 42.9 | 1.000 |
| Methadone in urine | 45.0 | 45.8 | 0.910° | 24.1 | 28.6 | 1.000 |
| Use street methadone | 37.2 | 29.2 | 0.275° | 20.8 | 15.4 | 1.000 |
| HIV seropositive | 42.0 | 60.4 | 0.015°* | 37.9 | 42.9 | 1.000 |
| Viral Load >1000 cp/mL | 16.6‡ | 13.8 | 1.000 | 20.0† | 83.3 | 0.035* |
| HCV seropositive | 74.9 | 77.1 | 0.738° | 58.6 | 85.7 | 0.095 |

Abbreviations: HSV-2, Herpes simplex virus 2; VND, Viet Nam Dong; HIV, Human Immunodeficiency Virus; cp/mL; copies per milliliters; HCV; Hepatitis C Virus. All p-values were obtained using Fischer’s exact test for categorical variables and Wilcoxon’s ranksum for continuous variables. °Denotes a p-value obtained using Pearson’s Chi-squared. *Denotes a statistically significant p-value. ^1^composite variable if answered “yes” to has ever smoked meth or has ever injected meth, and declares smoking or injecting at least once in past month or MET was present in urine. †1 missing value, ‡3 missing values.

**Supplementary Table S4. Sensitivity analysis including declared sexual behavior variables, for risk factors for HSV-2 seroconversion from whom a second HSV-2 test was performed, by gender**

| Characteristic at baseline | Crude IRR [95%CI] | aIRR [95%CI] | p-value |
| --- | --- | --- | --- |
| Men PWID, n=485 |  |  |  |
| HIV status |  |  |  |
| *Positive* | 1.9 [1.1-3.4] | 2.3 [1.3-4.2] | 0.006 |
| Having been injecting heroine |  |  |  |
| *< 5 years* | Ref. | Ref. |  |
| *5 to <10 years* | 1.7 [0.9-3.4] | 2.5 [1.2-5.0] | 0.012 |
| Reported never having sexual intercourse in lifetime | 0.5 [0.2-1.2] | 0.5 [0.2-1.1] | 0.067 |
| Women PWID, n=43 |  |  |  |
| Reported sex for money | 2.4 [0.8-7.0] | 8.7 [2.2-34.7] | 0.002 |
| Reported never having sexual intercourse in lifetime | 7.1 [2.0-25.5] | 16.4 [3.1-89.4] | 0.001 |

Both multivariate models are also adjusted for age and income. Abbreviations: HSV-2, Herpes simplex virus 2; PWID, People who inject drugs; HIV, Human immunodeficiency virus.
